# Using an Active-Optical Sensor to Develop an Optimal NDVI Dynamic Model for High-Yield Rice Production (Yangtze, China)

**DOI:** 10.3390/s17040672

**Published:** 2017-03-24

**Authors:** Xiaojun Liu, Richard B. Ferguson, Hengbiao Zheng, Qiang Cao, Yongchao Tian, Weixing Cao, Yan Zhu

**Affiliations:** 1 National Engineering and Technology Center for Information Agriculture, Key Laboratory for Crop System Analysis and Decision Making, Ministry of Agriculture, Jiangsu Key Laboratory for Information Agriculture, Jiangsu Collaborative Innovation Center for Modern Crop Production, Nanjing Agricultural University, Nanjing 210095, China; liuxj@njau.edu.cn (X.L); 2015201019@njau.edu.cn (H.Z.); qiangcao@njau.edu.cn (Q.C.); yctian@njau.edu.cn (Y.T.); caow@njau.edu.cn (W.C.); 2 Department of Agronomy and Horticulture, University of Nebraska-Lincoln, Lincoln, NE 68583, USA; rferguson1@unl.edu

**Keywords:** sensor, rice, high-yield, NDVI, model

## Abstract

The successful development of an optimal canopy vegetation index dynamic model for obtaining higher yield can offer a technical approach for real-time and nondestructive diagnosis of rice (Oryza sativa L) growth and nitrogen (N) nutrition status. In this study, multiple rice cultivars and N treatments of experimental plots were carried out to obtain: normalized difference vegetation index (NDVI), leaf area index (LAI), above-ground dry matter (DM), and grain yield (GY) data. The quantitative relationships between NDVI and these growth indices (e.g., LAI, DM and GY) were analyzed, showing positive correlations. Using the normalized modeling method, an appropriate NDVI simulation model of rice was established based on the normalized NDVI (RNDVI) and relative accumulative growing degree days (RAGDD). The NDVI dynamic model for high-yield production in rice can be expressed by a double logistic model: $\mathrm{RNDVI}={\left(1+e^{-15.2829\times \left(RAGDD_{i}-0.1944\right)}\right)}^{-1}-{\left(1+e^{-11.6517\times \left(RAGDD_{i}-1.0267\right)}\right)}^{-1}$ (R2 = 0.8577**), which can be used to accurately predict canopy NDVI dynamic changes during the entire growth period. Considering variation among rice cultivars, we constructed two relative NDVI (RNDVI) dynamic models for Japonica and Indica rice types, with R2 reaching 0.8764** and 0.8874**, respectively. Furthermore, independent experimental data were used to validate the RNDVI dynamic models. The results showed that during the entire growth period, the accuracy (k), precision (R2), and standard deviation of RNDVI dynamic models for the Japonica and Indica cultivars were 0.9991, 1.0170; 0.9084**, 0.8030**; and 0.0232, 0.0170, respectively. These results indicated that RNDVI dynamic models could accurately reflect crop growth and predict dynamic changes in high-yield crop populations, providing a rapid approach for monitoring rice growth status.

## 1. Introduction

The growth and nutrient indicators of crop types have their appropriate threshold ranges. To achieve the target grain yield and quality, the shoot biomass, leaf area index (LAI), and plant nitrogen (N) concentration must be at the correct threshold ranges [1,2,3]. Furthermore, the indicator threshold during the crop’s critical growth period can be used in real-time nutrition diagnosis, and can guide the quantitative application of fertilizer.

In recent years, with increased research on crop growth models, numerous scholars have concentrated on simulating the crop group tiller number and LAI, as well as other dynamic indicators [4,5,6,7,8,9,10,11,12]. Some LAI models of major cereal crops have established, for example, the universal LAI model and optimum LAI piecewise exponential model with the growth period as a driving factor [4,5]. Other drivers included the LAI linear model and logistic curve correction model set at accumulated temperatures [6,7]. On this basis, the universal LAI model was constructed with the relative accumulated temperature as a driving factor [8]. Furthermore, a normalization method considering the accumulated temperature and LAI was adopted to establish a normalized model [9] of summer maize on the Loess Plateau. In the dynamics of dry matter (DM) accumulation, Wang et al. [10] also established a logistic correction model that could dynamically simulate the DM accumulation. However, the applicability and reliability of the model relative to different varieties and different yield levels requires improvement. Relative research showed that differences due to the varieties and production levels can be eliminated by normalizing the experimental data before modeling. Moreover, the dynamic models should be universalized according to the crop growth index [11,12], although labor- and time-consuming destructive sampling is necessary to obtain the crop LAI, DM, N content, and other growth parameters.

In the last decade, remote sensing has been widely applied in agriculture. Crop growth indicators can be rapid, real-time, non-destructive, and accurately monitored by using vegetation indices (e.g., NDVI, radio vegetation index (RVI), difference vegetation index (DVI)). Among these vegetation indices, NDVI is a key indicator used in remote sensing that utilizes the low reflectivity of the red spectrum and the high reflectivity of the near-infrared spectrum. This vegetation index can characterize the growth status, biochemical characteristics, and coverage density evaluation, and has been widely used in crop identification and yield forecasting [13]. Numerous studies have reported that the spectral index at specific periods can be used to guide crop precision fertilization and predict grain yield [14,15]. For example, Franzen and Lukina [16,17] gathered the crop canopy NDVI and constructed the N fertilization optimization algorithm (NFOA) based on crop N uptake and yield, and improved the applications on sorghum, maize, and other crops [18,19]. The spectral index at the panicle initiation stage is also used to improve subsequent panicle fertilizer application in rice (Oryza sativa L.) [20,21]. Raun et al. [22] developed the concept of the response index (RI) and potential yield based on the NDVI value gathered by GreenSeeker^®^ (Trimble Navigation Limited, Sunnyvale, CA, USA) and the target yield of wheat in each stage. In consideration of the wheat yield at the final stage to recover the N response, Raun et al. [18] obtained an estimation of wheat production and constructed the N requirement amount algorithm. Instead of using the RI approach, Franzen et al. [23] used surface soil texture and tillage categories to refine sensor-based N recommendations. Further research was carried out to improve sensor-based yield estimation by using crop height measured via acoustic sensor [24]. Xue et al. [25,26] calculated the sufficiency index (SI) of early rice as the NDVI ratio of N deficiency treatment vs. an adequate treatment in Jiangxi Province, China. The normalization approach was used to construct a N regulation algorithm based on the SI and NDVI in rice. Canopy spectral indices better monitored crop growth to allow improved stewardship of the N topdressing during later growth stages. These spectral indices–based models for estimating crop growth status are easily affected by the crop variety and sensor type, etc. As such, the versatility and universality of these estimation models are subject to certain restrictions. If crop parameters are set as a monitoring factor, the nutrition status should be diagnosed and regulated by simulating the dynamic changes of the canopy spectral index in crop groups. Until now, few studies have been reported to establish a dynamic model based on spectral indices [27,28]. Carrying out further research on the variation in the spectral index and establishing a spectral index–based dynamic model is necessary for late crop nutrition monitoring and dynamic diagnosis.

Active canopy spectroscopy has the advantage of operating simply and non-destructively without being affected by weather conditions or the solar elevation angle. It can be used to quickly gather the canopy NDVI and other spectral indices of crops. Among the various active spectrometers, the most widely used sensor is GreenSeeker at two wavelengths (red, 656 ± 10 nm and near-infrared (NIR), 774 ± 10 nm), which is widely used to monitor crop growth and nutrient conditions. For example, Osborne [29] uses GreenSeeker to monitor wheat growth and nitrogen conditions, finding that NDVI values significantly correlated with dry matter, N content and accumulation. 

The first objective of this research was to perform a comprehensive analysis of the quantitative relationship of LAI, DM and grain yield (GY) with the canopy NDVI during the key growth period in rice. The second objective was to establish an appropriate rice canopy NDVI dynamic model for high-yield production by using the data normalization method. This NDVI dynamic model for obtaining high-yield production will provide a theoretical basis for precision agriculture.

## 2. Materials and Methods

### 2.1. Experimental Details

Experiments were conducted with varied N rates (0–375 kg·N·ha^–1^) in five Japonica rice and three Indica rice cultivars at Nanjing Experimental Station (31°56′ N, 118°59′ E) and Rugao Experimental Station (32°14′ N, 120°19′ E) in Jiangsu Province. The soil type at Nanjing Experimental Station is yellow soil, with soil total N content of 1.26 g·kg^–1^, available N content of 86.16 mg·kg^–1^, organic matter content of 18.4 g·kg^–1^, available phosphorus (P) content of 12.1 mg·kg^–1^, and available potassium (K) content of 79.6 mg·kg^–1^. The soil type at Rugao Experimental Station is loam, with a total N content of 1.66 g·kg^–1^, available N content of 96.23 mg·kg^–1^, organic matter content of 12.95 g·kg^–1^, available P content of 13.6 mg·kg^–1^, and available K content of 92.6 mg·kg^–1^. Detailed information is shown in Table 1.

In this study, we implemented five rice field experiments using a randomized block design and three replications. Experimental cultivars, sites, and N levels were presented in Table 1. P and K were applied in the beginning as basal fertilizer (135 kg·ha^–1^ P_2_O_5_, 203 kg·ha^–1^ K_2_O). Among the data sets, the data from experiments 3 and 4 were used to develop the NDVI dynamic model. According to the reference data for rice planting in Jiangsu Province [30], rice yield is divided into three production levels: low-yield group (yield ≤ 8.25 t·ha^–1^) with N application amounts of 0 kg·ha^–1^ and 75 kg·ha^–1^; middle-yield group (8.25 t·ha^–1^ < yield < 10.5 t·ha^–1^), with N application amounts of 150 kg·ha^–1^ and 225 kg ha^–1^; high-yield group (yield ≥ 10.5 t·ha^–1^) with N application amounts of 300 kg·ha^–1^ and 375 kg·ha^–1^. The data from experiments 1, 2, and 5 were used to validate these models. The corresponding N levels to the high-yield group in these experiments were 330 kg·ha^–1^, 360 kg·ha^–1^, and 330 kg·ha^–1^, respectively.

### 2.2. Sample Collection and Measurement

Rice canopy spectra were measured using a handheld GreenSeeker^®^ optical sensor (Trimble Navigation Limited, Sunnyvale, CA, USA), which measures near-infrared (780 ± 6 nm) and red light (671 ± 6 nm) bands. The NDVI and relative NDVI (RNDVI) were calculated using the measured values of the above bands. All measurements were taken on sunny days with no wind or breeze. The carried sensor probe was passed over the crop at a height of approximately 0.9 m above the crop canopy. Each cell consisted of three rows, and each row had a measurement of five replications. The analysis revealed that the spectral data varied significantly, with NDVI measurements based on the average value.

Synchronized with spectral measurements, continuous sampling was taken for five hills in each plot. Green leaf area was obtained using an LI-3000 portable area meter (Li-Cor, Lincoln, NE, USA) and expressed as LAI. Samples were oven-dried for 30 min at 105 °C to quickly cease plant metabolic activities and then at 70 °C at constant weight to attain the plant DM (t·ha^−^^1^). 

Grain yield was determined in each plot by harvesting plants manually from three randomly identified areas of 1 m^2^. Spikelets were removed from panicles and final grain yields were adjusted to 13.5% and 14.5% moisture content for Japonica and Indica rice, respectively. 

### 2.3. Data Processing and Model Construction

The data was normalized after the maximum conversion ratio method using 1st Opt.pro V1.5 (7D-Soft High Technology Inc., Beijing, China) and Origin.pro 7.5 fitting software (Origin Lab Corporation, Northampton, MA, USA). The curve was drawn with Microsoft Excel 2013 (Microsoft, Redmond, WA, USA). The analysis of variance (ANOVA) was done using IBM SPSS 20.0 software (IBM Corporation, Armonk, NY, USA).

(1) $${\mathrm{RAGDD}}_{i}={\mathrm{AGDD}}_{i}/{\mathrm{AGDD}}_{h}$$

(2) $${\mathrm{RNDVI}}_{i}={\mathrm{NDVI}}_{i}/{\mathrm{NDVI}}_{\max}$$

where RAGDDi is the relative accumulative growing degree days (GDD) on the i-th day after transplanting (i is the number of testing days after transplanting). AGDDi is the accumulative GDD on the i-th day after transplanting. AGDD_h_ is the accumulated GDD from transplanting to harvest. RNDVIi is the relative NDVI on the i-th day after transplanting. NDVIi represents the NDVI measured on the i-th day after transplanting. NDVI_max_ is the maximum NDVI of the same treatment or yield level during the entire growth period, obtained from historical or experimental data.

### 2.4. Model Validation

Model validation was conducted using the dataset from experiments 1, 2 and 5. The R^2^ and root mean square error (RMSE) were used to estimate the predictive accuracy of the model.

(3) $$\mathrm{NDVIs}\left(i\right)={\mathrm{NDVI}}_{\max}\times \mathrm{RNDVIs}\left(i\right)$$

(4) $$\mathrm{RMSE}=\sqrt{\frac{1}{n}\times \overset{n}{\underset{i=1}{\sum }}{\left(P_{i}-Q_{i}\right)}^{2}}$$

where NDVIs(i) is the simulated NDVI value on the i-th day after transplanting. RNDVIs(i) is the relative NDVIs(i) on the i-th day derived from the models. RMSE is the root mean square error, where n is the number of samples, P_i_ is the model simulated value derived from the models, and O_i_ is the observed value.

## 3. Results

### 3.1. Dynamic Changes of the Rice Canopy NDVI

**Figure 1:**
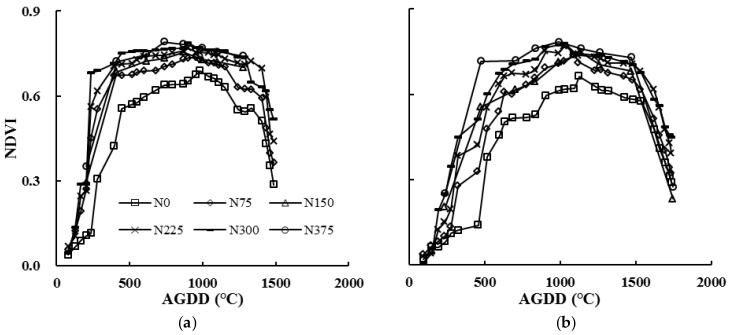
Time series changes of NDVI value for two rice cultivars used in experiment 4 with N rates of 0, 75, 150, 225, 300, 375 kg N·ha^−1^. (**a**) Indica (YLY-1); (**b**) Japonica (WYJ-24).

Experiment 4 was taken as an example to analyze the dynamic characteristics of the canopy NDVI during the entire growth period (Figure 1). Rice canopy NDVI values had a similar tendency, although they varied according to the cultivars and N levels. The NDVI values increased rapidly before reaching a plateau, then slowly when near the plateau. The values remained stable after reaching the plateau before a gradual decline. The varieties of both rice types achieved maximal NDVI values at the booting stage. At the early growth stages, because of the unclosed rice canopy, the bare soil and water background affected the canopy spectral sensor, reducing the reliability of using the NDVI for the estimation of crop growth indices. From the tillering stage, the NDVI value gradually showed a steep trend along with a rapid growth curve. With a more upright structure and weaker tillering capability, the Japonica rice has lower LAI and above-ground DM values compared with Indica rice, which resulted in the lower NDVI value in Japonica rice at the same yield level. After the booting stage, NDVI values were relatively stable below the maximum value for all cultivars and N treatments. Thus, a plateau appeared, and then a gradual downward trend occurred after the heading stage.

In addition, as shown in Table 2, the Indica rice usually has a larger maximum NDVI value than the Japonica rice under the same N level, and the Indica rice tends to reach the maximum NDVI value earlier than the Japonica rice. In Japonica rice, the maximum NDVI value increased along with the increasing N rates, and the NDVI of the higher N rate treatments reached the maximum value earlier than the lower N rate treatments. Therefore, an additional N application can increase the maximum NDVI value and hasten the attainment of the maximum NDVI value. Under the same conditions, Indica rice can easily achieve a greater maximum NDVI value in a shorter time period after transplanting because of its morphological structure.

### 3.2. Quantitative Relationships between the Rice Canopy NDVI and Population Growth Indices

The quantitative relationships of LAI, above-ground DM and GY with the canopy NDVI were analyzed at key growth stages, namely the jointing, booting, and heading stages, based on experiments 3 and 4.

#### 3.2.1. Quantitative Relationship between NDVI and LAI

**Figure 2:**
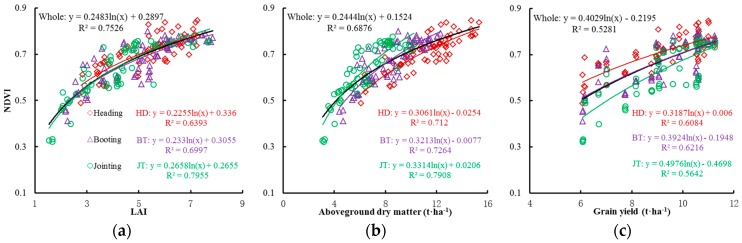
Quantitative relationships of NDVI to LAI (**a**), above-ground dry matter (**b**) and grain yield (**c**) in rice under varied N rates at jointing (JT), booting (BT) and heading (HD) growth stages.

With the advancement of the growth process, the increasing rate of the rice LAI kept pace with the canopy NDVI. The correlation between them, however, declined as the growth process moved forward (Figure 2a). This is mainly due to the gradual emergence of the panicle at the heading stage, leading to affected canopy spectra and NDVI values. With the increasing number of leaves, the shade effect becomes evident. The leaves at lower positions on the main stem and tillers cannot be well recognized by the canopy sensor, so during a fitting analysis, the NDVI value increases slowly or occurs with increasing LAI at the heading stage.

#### 3.2.2. Quantitative Relationship between NDVI and DM

Like LAI, DM is an important indicator of growth. Across growth stages, the canopy NDVI increased as the above-ground DM increased in rice, quickly at the jointing stage but then relatively slowly (Figure 2b). The above-ground DM and canopy NDVI values showed a good correlation, with R^2^ ranging from 0.71 to 0.79, and explained 68.76% of the NDVI variability during the entire growth period. The relationship between the NDVI and DM was relatively stable at different growth stages.

#### 3.2.3. Quantitative Relationship between NDVI and GY

The quantitative relationships were analyzed between the rice canopy NDVI and grain yield at key growth stages (Figure 2c). The results showed that the rice canopy NDVI was significantly positively related with the grain yield (GY) from the jointing to heading stages, with a similar relationship between the NDVI and above-ground DM (Figure 2b. The R^2^ values of three different stages ranged stably from 0.56 to 0.62. At the booting stage, the yield was most reliably estimated using the NDVI value.

### 3.3. Selection of RNDVI Dynamic Model

The AGDD, NDVI, and other agronomic indices of high-yield groups (yield ≥ 10.5 t·ha^–1^) were normalized in experiment 3 and experiment 4. Ten fitting equations between the RAGDD and RNDVI were established for selecting optimal models, including a double logistic curve, a rational equation, and a cubic polynomial equation. Table 3 lists the five well-fitted model types between RAGDD and RNDVI. Among them, the best determination coefficients (R^2^) of the double logistic, cubic polynomial, and rational equations were 0.8577**, 0.8357**, and 0.8319**, respectively.

**Figure 3:**
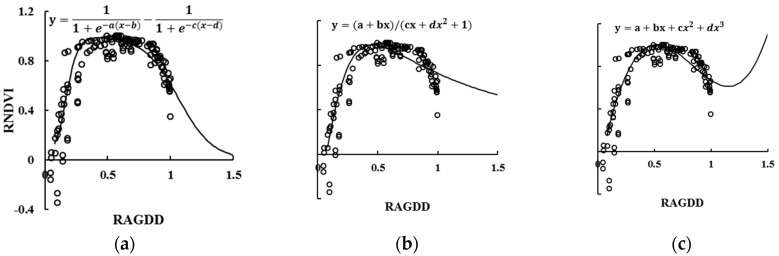
Three types of model for RNDVI dynamics with RAGDD: (**a**) the double logistic model; (**b**) the rational equation; (**c**) the cubic polynomial equation.

Figure 3 showed the results of the further analysis of the above three models. A good correlation of the three fitting equations can simulate the dynamic changes of the RNDVI for the high-yield rice population. However, for the polynomial equation, when x tends toward ∞, the simulated value of the RNDVI does not match the actual variation. The rational equation, which falls obviously from the booting to heading stages, cannot express the high plateau period of the NDVI curve well in rice during the same growth period. In contrast, the double logistic equation can simulate the RNDVI with the dynamics of RAGDD (Equation (5)).

(5) $$RNDVI={\left(1+e^{-a\times \left(RAGDD_{i}-b\right)}\right)}^{-1}-{\left(1+e^{-c\times \left(RAGDD_{i}-d\right)}\right)}^{-1}$$

where RAGDDi indicates the relative AGDD value on the i-th day after transplanting, and RNDVI corresponds to the relative NDVI value during the same period. Here, a and c are the crop’s two inflection points in the growth and senescence logistic curves, while b and d are the two time points, expressed as the RAGDD corresponding to the critical conversion time of a and c, respectively. 

### 3.4. Establishment of the RNDVI Dynamic Models 

**Figure 4:**
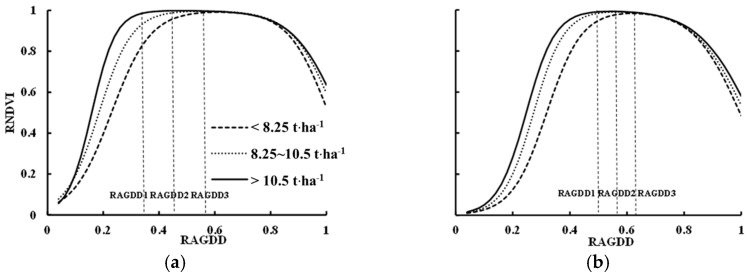
Comparisons of the double logistic simulated models of Indica (**a**) and Japonica (**b**) rice types based on different yield levels. RGDD1, RGDD2, and RGDD3 refer to the different RAGDDs with RNDVI peaks.

The diversity of plant morphology exists due to different rice cultivars. To further improve the forecast accuracy of the RNDVI dynamic model, models of Japonica and Indica rice under different production levels were constructed using the double logistic method (see Figure 4). With the relevant parameters shown in Table 4, the coefficients of determination (R^2^) of the NDVI dynamics in the two rice types were all above 0.86. Furthermore, a significant test was performed between these model parameters (Table 4) and the double logistic model’s parameters (Table 3). The results demonstrated that all values were non-significant at the 5% level (i.e., the resulting *t*-values were >0.05). Thus, the relative dynamic model of the NDVI can be used to accurately simulate the NDVI dynamics of rice high-yield groups.

The RNDVI dynamic models of *Indica* (RNDVI_In_) and *Japonica* (RNDVI_Ja_) rice are as follows:

(6) $$RNDVI_{In}={\left(1+e^{-23.8261\times \left(RAGDD_{i}-0.1489\right)}\right)}^{-1}-{\left(1+e^{-12.0923\times \left(RAGDD_{i}-1.0361\right)}\right)}^{-1}$$

(7) $$RNDVI_{Ja}={\left(1+e^{-20.0313\times \left(RAGDD_{i}-0.2370\right)}\right)}^{-1}-{\left(1+e^{-10.9741\times \left(RAGDD_{i}-1.0195\right)}\right)}^{-1}$$

RNDVI dynamic models constructed at different yield levels for both Indica and Japonica rice showed a similar tendency (Figure 4). However, some differences still existed in the simulated dynamics of the NDVI. The greater the yield potential was, the higher the increasing rate of the NDVI before reaching the peak value, the longer the NDVI peak plateau, and the lower the decreasing rate of the NDVI after the peak value. This resulted in the higher value of parameter a, the lower value of parameter b and c, and the higher parameter d.

**Figure 5:**
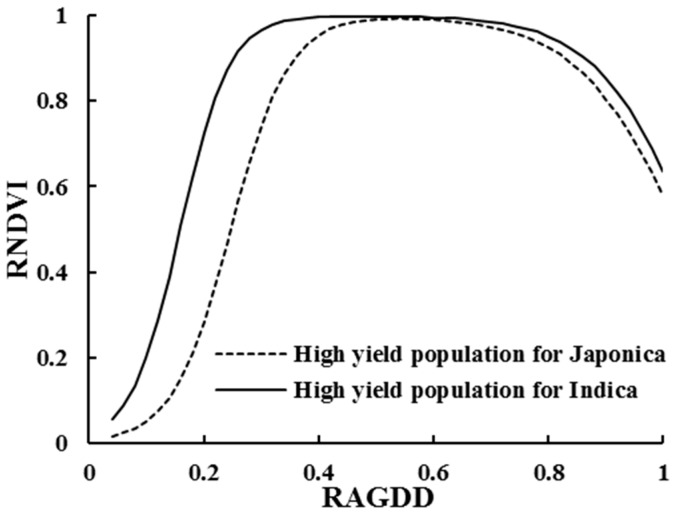
Comparison of the RNDVI dynamics of Indica and Japonica rice types under the high-yield level (yield > 10.5 t·ha^−1^).

Figure 5 shows the differences in RNDVI changes for different rice types. A large difference between the two rice types occurred at the early growth stage; in particular, the growth rate of the RNDVI in Indica rice was faster than that in Japonica rice, and the Indica rice reached the double logistic curve inflection point earlier than the Japonica rice did, mainly due to the Indica’s plant structure. At the same RAGDD, Indica rice achieved higher RNDVI values because of the loose leaf morphology, and higher LAI and DM. The differences in the maximum RNDVI value between the two types of rice were eliminated by using the data normalization method, which is reflected in the growth rate of the RNDVI. After a stable plateau, rice populations entered the aging period, and the aging trend of the two types of rice was quite similar. The time inflection point (d value), the RNDVI decline rate (c value), and the maximum rate of decrease were not significantly different.

### 3.5. Model Validation

**Figure 6:**
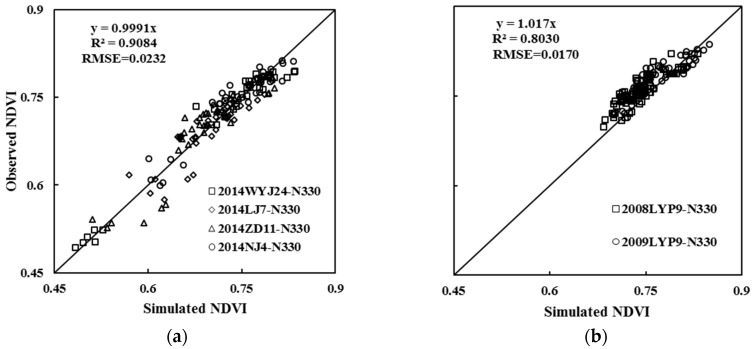
The relationships between the observed and simulated NDVI values of two rice cultivars (**a**) Japonica, (**b**) Indica, from tillering growth stage to flowering growth stage. The solid line is inclined at 45° to the axes.

Independent observed NDVI values were used to validate the NDVI dynamic models for Indica and Japonica rice under the high-yield level. Simulated NDVI values from 15 July (tillering stage) to 6 September (heading stage) under the high-yield level were calculated by the models with RAGDD as a driving factor. The validation result is shown in Table 5, where the k value was between 0.9692 and 1.0330, and all were around 1, and R^2^ (representing the simulation accuracy, as below) was between 0.6102 and 0.9331, reaching a highly significant level. The RMSE values between the observed and simulated NDVI values were between 0.0064 and 0.0305, indicating high simulation accuracy. From the accuracy and precision of the simulation results, the pre- and post-simulation results better reflected the changes in the population dynamics. The comprehensive comparisons of simulated and observed values over entire growth stages revealed that the k values of Japonica and Indica rice were 0.9991 and 1.0170, respectively; the R^2^ values were 0.9084 and 0.8030; and the RMSE were 0.0232 and 0.0170 (Figure 6). As the sample number increased, the fitting effect throughout the growth period also reached a high degree of accuracy. Results indicated the RNDVI dynamic models could accurately reflect and simulate the population growth dynamics of high-yield rice.

## 4. Discussion

Population growth indices such as LAI and DM can effectively designate crop growth conditions [30,31]. Remote sensing provides a new approach to monitor growth indices, nutrition indices and yields of crops [32,33]. GreenSeeker active-optical sensors effectively assess crop growth conditions and facilitate post-assessment of the N requirement. This also has the advantage of operating simply and non-destructively, rendering it superior to other gathering methods or indicators [34,35,36,37]. Different crop cultivars and N levels can lead to different LAI values [38]; Indica rice normally has higher LAI values than Japonica rice due to its loose leaf morphology. This study found that rice canopy NDVI values had a similar tendency during the entire growth period, and both achieved maximal NDVI values at the booting stage. In addition, researchers conducted several experiments on maize, rice and wheat, and found that the spectral index can be used to monitor crops’ main growth indices [39,40,41,42,43,44]. This confirmed, to some extent, the stable relationships of the canopy NDVI with LAI, above-ground DM and GY performed at key growth stages (Figure 2). Furthermore, we found that the NDVI became saturated when the value was >0.7, a slight variation on this value compared to Goswami’s result [45]. This is due to different crop varieties and eco-sites.

When previous dynamic models based on crop growth indicators and spectral indices are applied for crop production, destructive sampling may be needed to obtain the LAI and other agronomic parameters. The simulation could proceed based on growing degree days, or a sub-function simulation may be required, which restricts the applications of these models [4,5,11,45] to some extent. In this study, the RNDVI models based on RAGDD were constructed for both Japonica and Indica high-yield rice types. The accuracy of the model reached 0.86, and the accuracy of the RNDVI model fitted separately on Indica and Japonica rice also reached 0.89 and 0.88, respectively. The normalization of the data can weaken the effects of cultivars and other factors on the model, allowing common trends in crop production. By taking crop growth into consideration, and fully analyzing the curve characteristics and range of each model, only the double logistic curve could properly express and simulate the canopy RNDVI dynamic characteristics for high-yield rice groups. This includes rapid growth after tillering, the stable plateau during booting, and a slow decline after heading. Furthermore, accumulative GDD was selected as a model variable parameter, and the AGDD for the similar crop growth period had a determined threshold range. These parameters promoted the use of the RNDVI dynamic model in production and practice. Therefore, the present study compensates for the weakness of previous models with a complex structure or more input parameters requiring destructive sampling. This study used an active-optical sensor to explore a rapid and convenient method for acquiring the dynamic changes of crop NDVI. The model has the advantages of a simple structure, fewer input parameters, and ease of use.

In addition, experiments using N levels were only used on five rice cultivars in the present study. Moreover, experiment sites were also limited to the Yangtze River area, where cultivar types and eco-sites are relatively homogeneous. These factors may affect model parameters, reducing the universality of the model [11]. Further study is required to solve these problems. At the same time, in order to deeply explain the NDVI dynamic characteristics under the crop high-yield level and to regulate top-dressing fertilizer, the relationships among the model parameters, biomass production, LAI, N accumulation, unit tiller number, and other growth indicators at different levels are still required for accurate quantification. Furthermore, more experimental data is required to evaluate and calibrate the model in the future.

## 5. Conclusions

The canopy NDVI has a relatively stable and positive relationship with rice growth indices such as the LAI, above-ground dry matter and grain yield, etc. By using the normalizing method, the canopy RNDVI dynamic model based on accumulative growing degree days was constructed for high-yield production of rice in the Yangtze River region. Furthermore, the variation among different cultivars was taken into consideration, requiring the construction of relative NDVI (RNDVI) dynamic models for Japonica and Indica rice types, with R^2^ reaching 0.8764** and 0.8874**, respectively. These results indicate that RNDVI dynamic models can accurately reflect crop growth and predict dynamic changes in high-yield crop populations, providing a rapid and non-destructive approach for diagnosing rice growth status and formulating N nutrition diagnosis. This paradigm can be used to model and predict the canopy NDVI for obtaining high-yield production in rice, which also provides a technical pattern for real-time diagnosis of crop growth and nutrition conditions.

## Figures and Tables

**Table 1:** Basic information about five field experiments.

Experiment NO.	Location	Cultivar	N Rate (kg·ha^–1^)	Plot Size (m^2^)	Transplanting Date (Month/Day)	Sampling Date (Month/Day)
EXP. 1 in 2008	Nanjing	LYP9 (Indica)	0, 110, 220, 330	4.5 × 6.5 = 29.25	6/23	07/16, 07/20, 07/26, 07/30, 08/03, 08/08, 08/13, 08/18, 08/23, 08/28, 08/31, 09/05
EXP. 2 in 2009	Nanjing	LYP9 (Indica)	0, 180, 360	5.0 × 6.0 = 30.0	6/17	07/15, 07/19, 07/25, 07/30, 08/04, 08/07, 08/13, 08/17, 08/22, 08/27, 09/02, 09/06
EXP. 3 in 2013	Rugao	WXJ14 (Japonica)	0, 75, 150, 225, 300, 375	5.0 × 6.0 = 30.0	6/22	06/28, 07/03, 07/08, 07/11, 07/19, 08/08, 08/12, 08/15, 08/19, 08/25, 09/17, 09/21, 09/25, 10/02, 10/10, 10/11
		SY63 (Indica)				
EXP. 4 in 2014	Rugao	WXJ24 (Japonica)	0, 75, 150, 225, 300, 375	6.0 × 7.0 = 42.0	6/17	06/23, 06/27, 06/30, 07/03, 07/06, 07/09, 07/17, 07/20, 07/24, 07/26, 07/29, 08/03, 08/06, 08/10, 08/16, 08/19, 08/23, 08/25, 08/30, 09/02, 09/05, 09/08, 09/16, 09/21, 09/25, 10/02, 10/06, 10/10, 10/14
		YLY1 (Indica)				
EXP. 5 in 2014	Rugao	WYJ24 LJ7 ZD11	0, 110, 220, 330	5.0 × 6.0 = 30.0	6/17	7/18, 7/30, 08/06, 08/16, 08/26, 09/04
		NJ4 (Japonica)				

Rice cultivar: Wuxiangjing-14 (WXJ14), Wuyunjing-24 (WYJ24), Ningjing-4 (NJ4), Lianjing-7 (LJ7), Zhendao-11 (ZD11), Liangyoupei-9 (LYP9), Shanyou-63 (SY63), Y liangyou-1 (YLY1).

**Table 2:** The NDVI_max_ value, days after transplanting (DAT) and AGDD when obtaining the NDVI_max_ value for different rice cultivars and N treatments in experiments 3 and 4.

Items		NDVI_max_				DAT(d)/AGDD (°C) When Obtaining the NDVI_max_ Value			
Year		2013		2014		2013		2014	
Cultivar		WXJ14 (Japonica)	SY63 (Indica)	WYJ24 (Japonica)	YLY1 (Indica)	WXJ14 (Japonica)	SY63 (Indica)	WYJ24 (Japonica)	YLY1 (Indica)
N Treatment	0	0.489d	0.689c	0.663c	0.688c	65a/1285.5a	65a/1155.5a	70a/1123.5b	69a/983.5a
	75	0.632c	0.759b	0.737b	0.736b	65a/1285.5a	59ab/1052b	68b/1092.5c	67b/956.5b
	150	0.645bc	0.870a	0.746b	0.754ab	59b/1170b	59ab/1052b	70a/1139a	60d/869.5d
	225	0.659b	0.880a	0.769a	0.769ab	59b/1170b	59ab/1052b	64c/1030.5d	63c/902.5c
	300	0.660b	0.886a	0.777a	0.787a	55c/1089.5c	55b/979.5c	64c/1030.5d	63c/902.5c
	375	0.691a	0.888a	0.784a	0.790a	55c/1089.5c	55b/979.5c	60d/991.5e	50e/736.5e

F-test statistical significance at 0.05 probability level.

**Table 3:** Coefficient of parameters, determination and RMSE of the RNDVI dynamic model with RAGDD.

Simulated Models	Parameters				R^2^	RMSE
	a	b	c	d		
$y={\left(1+e^{-a\times \left(x-b\right)}\right)}^{-1}-{\left(1+e^{-c\times \left(x-d\right)}\right)}^{-1}$	15.2829	0.1944	11.6517	1.0267	0.8577	0.1161
$y=a+\mathrm{bx}+{\mathrm{cx}}^{2}+{\mathrm{dx}}^{3}$	–0.3796	5.7851	–7.4437	2.7004	0.8357	0.1357
$y=\left(a+\mathrm{bx}\right)/\left(\mathrm{cx}+{\mathrm{dx}}^{2}+1\right)$	–0.4319	5.3826	–0.3948	6.1176	0.8319	0.1373
$y=\left(\mathrm{ab}+{\mathrm{cx}}^{d}\right)/\left(b+x^{d}\right)$	–0.1741	0.0010	0.8720	3.5469	0.7671	0.1616
$y=a/\left(1+{\mathrm{be}}^{-\mathrm{cx}}\right)$	0.8635	97.9447	27.3641	–	0.7549	0.1674

x is the RAGDD, y is RNDVI.

**Table 4:** Parameters of the RNDVI dynamic model based on different cultivars and N rates for three yield levels.

Cultivar Type	Yield Level	N Rate	Cultivar	Yield	NDVI_max_ Value	Entire Growing Period	Parameter	R^2^	RMSE
	(t·ha^–1^)	(kg·ha^–1^)		(t·ha^–1^)		(days)			
*Japonica*	Low (yield ≤ 8.25 t·ha^–1^)	0	WXJ14	6.08	0.489	150	a: 16.4599	0.8834	0.1370
			WYJ24	6.70	0.663	156	b: 0.3090		
		75	WXJ14	7.75	0.632	150	c: 12.3144		
			WYJ24	7.87	0.737	156	d: 0.9851		
	Middle (8.25 t·ha^–1^ < yield < 10.5 t·ha^–1^)	150	WXJ14	8.78	0.645	150	a: 19.0544	0.9024	0.1224
			WYJ24	8.98	0.746	156	b: 0.2629		
		225	WXJ14	9.08	0.659	150	c: 11.4756		
			WYJ24	9.62	0.769	156	d: 1.0022		
	High (yield ≥ 10.5 t·ha^–1^)	300	WXJ14	10.53	0.660	150	a: 20.0313	0.8764	0.1367
			WYJ24	10.54	0.777	156	b: 0.2370		
		375	WXJ14	10.61	0.691	150	c: 10.9741		
			WYJ24	10.63	0.784	156	d: 1.0195		
*Indica*	Low (yield ≤ 8.25 t·ha^–1^)	0	SY63	6.16	0.689	153	a: 14.3656	0.8713	0.1175
			YLY1	7.01	0.688	133	b: 0.2196		
		75	SY63	8.17	0.759	153	c: 14.0343		
			YLY1	8.20	0.736	133	d: 0.9972		
	Middle (8.25 t·ha^–1^ < yield < 10.5 t·ha^–1^)	150	SY63	9.04	0.870	153	a: 17.1028	0.8610	0.1128
			YLY1	9.25	0.754	133	b: 0.1749		
		225	SY63	9.86	0.880	153	c: 13.2413		
			YLY1	10.13	0.769	133	d: 1.0192		
	High (yield ≥10.5 t·kg·ha^–1^)	300	SY63	10.61	0.886	153	a: 23.8261	0.8874	0.0981
			YLY1	10.83	0.787	133	b: 0.1489		
		375	SY63	11.02	0.888	153	c: 12.0923		
			YLY1	11.26	0.790	133	d: 1.0361		

**Table 5:** Coefficient of k, determination (R^2^) and RMSE of the linear correlation between the observed and simulated NDVI values at different growth stages.

Growth Stage	k		R^2^		RMSE	
	Japonica	Indica	Japonica	Indica	Japonica	Indica
Active tillering	0.9749	1.0158	0.7044 **	0.6102 **	0.0305	0.0128
Middle tillering	1.0187	1.0318	0.7689 **	0.7990 **	0.0164	0.0075
Jointing	1.0045	1.0330	0.9331 **	0.6656 **	0.0079	0.0105
Booting	1.0160	1.0098	0.6565 **	0.7367 **	0.0191	0.0113
Heading	1.0086	0.9859	0.9167 **	0.8211 **	0.0174	0.0064
Flowering	0.9692	1.0333	0.8762 **	0.6357 **	0.0119	0.0128
Active tillering to flowering	0.9991	1.0170	0.9084 **	0.8030 **	0.0232	0.0170

** F-test statistical significance at 0.05 probability level.
